# A Qualitative Account of Young People’s Experiences Seeking Care from Emergency Departments for Self-Harm

**DOI:** 10.3390/ijerph18062892

**Published:** 2021-03-12

**Authors:** Sadhbh J. Byrne, India Bellairs-Walsh, Simon M. Rice, Sarah Bendall, Michelle Lamblin, Emily Boubis, Brianna McGregor, Meghan O’Keefe, Jo Robinson

**Affiliations:** 1 Orygen, Parkville, VIC 3052, Australia; india.bellairs-walsh@orygen.org.au (I.B.-W.); simon.rice@orygen.org.au (S.M.R.); sarah.bendall@orygen.org.au (S.B.); michelle.lamblin@orygen.org.au (M.L.); emilyboubis@hotmail.com (E.B.); mcgregor.briannalouise@gmail.com (B.M.); meghan.okeefe@hotmail.com (M.O.); jo.robinson@orygen.org.au (J.R.); 2 Centre for Youth Mental Health, The University of Melbourne, Parkville, VIC 3010, Australia; 3 Centre for Global Health, Trinity College Dublin, Dublin 2 D02 PN40, Ireland

**Keywords:** self-harm, emergency departments, young people, qualitative

## Abstract

Many young people who engage in self-harm do not seek help from health services. For those that do, emergency departments (EDs) are a key point of contact. Substantial gaps remain in current knowledge related to young consumers’ experiences and views on optimal treatment of self-harm in the ED. In this study, semi-structured interviews were conducted with thirteen young people (*M*_age_ = 21.2 years), who were engaged with care at headspace early intervention centers and had presented to an ED with a self-inflicted physical injury. Participants were asked to describe their experience in the ED and the care they received. Data were analyzed thematically. Three inter-related themes were identified: 1. The ED was experienced through a lens of significant distress, 2. The ED environment and processes were counter-therapeutic, and 3. Staff were perceived to be disinterested, dismissive, and lacking in knowledge. The study highlights the overwhelmingly negative nature of participants’ experiences, and presents recommendations for service and practice improvements, such as the provision of staff training and increased aftercare.

## 1. Introduction

Self-harm refers to a range of behaviors (including self-poisoning and self-injury) through which an individual directly causes harm to themselves, irrespective of motive, including the degree of suicidal intent [1,2]. This term is therefore inclusive of attempted suicide, as well as self-harm serving functions such as emotion regulation and self-punishment [3,4]. Self-harm typically emerges during adolescence and young adulthood, and is associated with adverse mental and physical health outcomes, social disadvantage, subsequent death by suicide and increased all-cause mortality [5,6,7,8,9,10]. Many young people do not seek help for self-harm from health services [11,12] but for those that do, emergency departments (EDs) are a key point of contact [13]. Presentation to an ED often marks the first instance in which a young person discloses their self-harm [14], and as such presents an opportunity to engage those at-risk and provide therapeutic interventions and/or link them to appropriate longer term supports [15].

Recent literature suggests the treatment of those who present to an ED with self-harm is often sub-optimal [16]. In one study, young participants reported feeling belittled and shamed, and some reported being more likely to engage in repeat self-harm after leaving the ED, due to these experiences [17]. Research on consumers’ experiences of self-harm care has predominantly focused on adults [18,19,20,21], and a recent systematic review revealed a substantial gap in knowledge of young consumers’ experiences and views on optimal treatment of self-harm in the ED [22]. This gap, and the notion that young people who self-harm are generally considered ‘vulnerable’ research participants [20], means that the acceptability, feasibility and safety of investigating young peoples’ experiences is undetermined. Young people have the right to exert their competence and express their views on matters that affect them [23,24]. As empowerment and user involvement are important tenets of contemporary mental health care, young people’s experiences and perspectives must play a central role in shaping best practice [25,26].

### The Current Study

The aims of the current study were to: 1. examine young consumers’ views on their experiences of seeking care for self-harm from the ED, and 2. examine the acceptability, feasibility and appropriateness of conducting research that asks young people to recount experiences of ED care for self-harm.

## 2. Materials and Methods

This study is reported in line with the COREQ guidelines—see Supplementary File S1 for checklist [27,28].

### 2.1. Study Design and Methodological Orientation

An exploratory mixed-methods design was used. Qualitative data were collected through face-to-face interviews using a semi-structured interview schedule, and quantitative data were collected to assess participants’ level of distress pre- and post-interview. Participants provided both quantitative and qualitative data through a purpose-designed set of questions on the recruitment and data collection methods.

This study’s qualitative inquiry is underpinned by phenomenology, with the aim of methodologically, carefully, and thoroughly describing how participants experienced their presentations to the ED. Phenomenological methods are often used to explore sensitive topics, as the goal is not only to record participants’ experiences, but to explore the meaning associated with these experiences [29,30]. The inherent subjectivity of participants’ accounts is acknowledged but is not considered a limitation [31,32]. This is well-aligned with the principles of person-centered care [33], which posits that an individual’s experience of healthcare can determine the outcome of said care. A secondary aim of this study was to establish participants’ views on how research on this topic should be conducted. Intrinsically, therefore, the study is oriented within the participatory framework, the purpose of which is to facilitate more empathic and democratizing approaches to research participation, particularly for people deemed ‘vulnerable’ [34].

### 2.2. Setting, Sample and Recruitment

The study was conducted by researchers at Orygen in Melbourne, Australia. Participants were recruited from four headspace centers across north and west metropolitan Melbourne. headspace is Australia’s national youth mental health foundation, and provides primary care to young people aged between 12 and 25 [35,36]. A project advisory group was established which included three youth advisors (E.B., B.M., and M.O.), a headspace staff representative, and a member of the emergency department mental health team.

The inclusion criteria for participation were developed through a review of the literature and consultations with the advisory group. These were: 1. Aged between 12–25 years; 2. Currently or recently engaged with one of the four participating headspace centers; 3. Had, at some point, presented to an ED with self-harm (with or without suicidal intent); 4. Able to provide informed consent. As caregiver consent was required for young people under 18 years, their caregiver must have been aware of the ED presentation.

Two researchers (S.J.B. and I.B.W.) provided information to headspace clinicians both face-to-face and via email. Potential participants were purposively selected by clinicians who approached the young person either in-person or over the phone, to give them initial information about the study and seek their permission to be contacted by a member of the research team.

Nineteen young people were referred to the study in total. One researcher (either S.J.B. or I.B.W.) contacted each potential participant by telephone, to provide information about the study and answer any questions. The same researcher then emailed a Participant Information and Consent Form (PICF—see Supplementary File S2) to be signed if they agreed to take part, together with caregiver consent for those aged under 18. Ultimately, 13 young people agreed to take part and provided written consent. Reasons for non-participation were not systematically collected.

### 2.3. Procedure

Interviews were conducted between May and July 2019, and were audio recorded. Each interview was conducted by both S.J.B. (Ph.D.) and I.B.W. (B.Psych.Sc. (Hons)). Both researchers identify as female, were employed as research assistants, and had previous experience conducting interviews and focus groups with young people. No relationship existed between the researchers and participants prior to study commencement. Each participant was interviewed in a quiet room at their regular headspace center. One researcher led the interview, while the other took notes. Interview length ranged from 34 min to 1 h and 12 min; the mean duration was 47 min.

#### 2.3.1. Demographics

Prior to the interview, participants completed a demographics questionnaire (see Supplementary File S3), which included age, gender identity, country of birth, Aboriginal or Torres Strait Islander status, and whether English was the main language spoken at home.

#### 2.3.2. Pre-Participation Distress

Prior to the interview, participants were asked to complete the Patient Health Questionnaire-4 item (PHQ-4) [37] as a pen-and-paper questionnaire. The PHQ-4 is an ultra-brief measure of depression and anxiety and served as an assessment of pre-participation distress. Participants rated how often they had experienced the problem described in each item on a four-point scale. Responses are scored as 0 (“not at all”), 1 (“several days”), 2 (“more than half the days”), or 3 (“nearly every day”). The reference period was the two weeks prior to interview. Scores were summed such that the potential range was 0–12, with higher scores indicative of greater distress. PHQ-4 scores have been categorized as normal (0–2), mild (3–5), moderate (6–8), and severe (9–12) [37]. 

#### 2.3.3. Interview Schedule

At the start of the interview, participants were asked about the number of presentations they had made to an ED for self-harm or a suicide attempt. Researchers advised participants who had made more than one presentation that they could choose to talk about one presentation in particular (e.g., their most recent presentation, or the presentation that they most clearly remembered) or talk about their cumulative experience. The interview schedule covered three main topics: 1. Starting at the point of your arrival, could you describe what happened during your time in the emergency department? 2. Were there any positive aspects to this experience? 3. Were there any negative aspects to this experience? Participants were also asked if they had any recommendations for service improvements. The specific interview schedule is appended in Supplementary File S4. This was reviewed by the youth advisors on the project, but was not formally pilot tested.

#### 2.3.4. Participation-Induced Distress

Post-interview, participants completed a pen-and-paper questionnaire which assessed participation-induced distress in absolute terms (i.e., participation was distressing) and in relative terms (i.e., participation was more distressing than everyday life events) using two sets of questions adapted from Yeater et al. [38].

#### 2.3.5. Feedback on Methodology

After completing the questionnaire on participation-induced distress, participants completed a final purpose-designed pen-and-paper questionnaire about the design of studies on this topic (see Supplementary File S5). Specifically, participants were asked about their views on the best time to contact a young person (i.e., how long after their ED presentation), the best way of contacting a young person, and the best way of asking a young person about their experiences in the ED. Space was also provided for participants to provide other suggestions.

### 2.4. Data Analysis

The audio recordings of interviews were transcribed by a professional transcription service; these were not returned to participants for comment. Transcripts were then imported to a qualitative software program (NVivo 12, QSR International, Melbourne, Australia, https://www.qsrinternational.com, accessed on 1 August 2019) to assist with data management and analysis. In line with the phenomenological orientation of the study, reflexive thematic analysis was conducted, with reference to Braun and Clarke [39,40]. This approach to analysis is well-suited to research questions related to people’s experiences, and importantly, recognizes a continuum between inductive and deductive analytic processes. The same researchers (S.J.B. and I.B.W.) conducted both data collection and analysis, and endeavored to ensure that analyses remained grounded in the data and to use participants’ words where possible.

S.J.B. familiarized and immersed herself in the data by reading and re-reading the transcripts and listening to the audio-recordings. The second step involved a systematic analysis of the data through generating initial codes and annotating transcripts. At this point, I.B.W. reviewed the data corpus by reading through transcripts with S.J.B.’s coding structure in place, while listening to the audio-recordings. Further coding and annotating of the data were conducted by I.B.W. at this stage, with the aim of developing a richer and more nuanced reading of the data. Data referring to any experience that occurred outside of the ED (e.g., interactions with paramedics whilst being transported to the hospital) were coded as ‘outside of scope’ and not included. Next, both researchers met to discuss the codes and began an iterative process of theme generation. The researchers inspected codes for contradictory or disconfirming cases. However, there were very few, if any, examples of incongruence; where these were identified, they are reported below. Participants did not provide feedback on the findings.

### 2.5. Safety and Ethics

The study was approved by The University of Melbourne’s Human Research Ethics Committee (Ethics ID: 1852466).

The secondary aim of this study was to understand the safest means by which to carry out research on this topic with young people. The study design was thus informed by careful consideration of any potential risks. The safety-related practices that were implemented are outlined in more detail in Supplementary File S6. In line with best practice guidelines [41,42], participants were reimbursed $30.00 per hour for their time.

## 3. Results

### 3.1. Demographic Information

The mean age of the sample was 21.2 years (SD = 2.1; range = 17–25 years). Eleven participants identified as female, and two identified as intersex, trans, gender fluid or gender diverse, with one of these two concurrently identifying as male. Eleven participants were born in Australia, one was born in the Philippines, and one in Fiji. No participants identified as Aboriginal or Torres Strait Islander. All participants reported that English was the primary language spoken at home.

Participants were asked about the number of presentations they had made to an ED for self-harm or a suicide attempt. Three participants said that they had presented once, while the other ten participants reported multiple presentations. One participant reported “over 50” presentations, and another reported a “10-year history” of presentations.

### 3.2. Describing Their Experience of the ED

Three inter-related themes were identified, encapsulating both participants’ experiences and, where relevant, their suggestions for improvements. These themes are presented in detail below.

#### 3.2.1. The ED Was Experienced through a Lens of Significant Distress

Participants’ accounts highlighted the impact of their emotional state at presentation on their experiences. Typically, participants had presented to the ED immediately after their episode of self-harm, which impeded their ability to navigate the service:

*“This major thing has just happened in your life and you can’t really comprehend anything at that point...”*

(P7)

It also meant that they were already distressed, exhausted, vulnerable, agitated, and “in the worst mindset” (P7). In some cases, this was further impacted by the specific method of self-harm used, with some participants still acutely affected by the substance(s) they had consumed, and reported feeling drowsy, “so out of it” (P6), “in a blurred state” (P9), and “in a haze” (P13). Participants discussed how their self-harm was prompted by, and subsequently exacerbated, negative feelings towards themselves, including anger and shame, which impacted on their ED presentations. Many expressed feeling embarrassed:

*“[I wish that EDs] could just have this, like, winking signal that [attendees could use to indicate] like, ‘Yeah, I just tried to kill myself’. That would be good, because it really is embarrassing to say ‘Hi, I just tried to kill myself’ and I’m here at a place that is designed to keep people alive.”*

(P9)

Participants felt “selfish and needy” (P8), particularly due to the self-inflicted nature of their injuries: “I always apologized because I felt bad that this was something that I did to myself and then they need to work around [me].” (P5). This led young people to feel undeserving of basic aspects of care, with one participant revealing, “I felt really bad for asking [for a cup of water] … because there were people suffering next to me.” (P11). Self-stigma was evidenced through participants’ language, for example referring to other attendees as “actually” (P8) unwell, implying that participants themselves, in contrast, were not. However, others experienced inner conflict:

*“You’re kind of fighting between two sides of, ‘I want to tell you that I need to be here’, but then you’re also fighting this urge in your head, like ‘Don’t be an attention seeker. You really aren’t that bad in comparison to everyone else here’.”*

(P9)

Overall, this impacted upon participants’ already-negative self-evaluations, and led them to second-guess whether they should have presented at all: “I was already really embarrassed about it and then I just felt like an idiot for even coming in.” (P5). Some participants stated this led them to leave the ED before they felt ready.

#### 3.2.2. The ED Environment and Processes Were Counter-Therapeutic

Participants indicated that not only did their experience in the ED fail to meet their needs, it also actively increased their distress. This commenced with the lack of privacy in the waiting room; participants recounted how triage staff spoke loudly, such that other ED attendees could overhear details of their presentation:

*“Everyone can hear what I’m saying and it’s like, well I don’t really want people to know. It’s not a thing that I like to talk about in public. ‘I’m suicidal, I’ve done this’.”*

(P10)

Some indicated that this lack of privacy affected how much information they disclosed to staff. As a solution, one participant suggested being able to complete an online questionnaire in the waiting room, describing what they were experiencing. Others suggested that privacy concerns could be addressed by allocating a specific area for mental health-related presentations, or by conducting triage assessments in a private area.

All participants reported extremely long waiting periods after their initial triage, some lasting up to 12 h. Rarely were participants given an indication of how long they should expect to wait, or the purpose of the wait, leading them to believe they were being “ignored” (P3) or “forgotten” (P11). Waiting was unpleasant due to the “stressful” (P2) and “scary” (P7) environment of the ED, with loud noises and bright lights, which was particularly impactful given participants’ emotional state at presentation. Those who had experience presenting to the ED both as a child and as an adult felt that the ED at the children’s hospital was less intimidating, such that “sometimes I wish I could go into the kids’ section, because adults are scary.” (P10). While waiting, participants attempted to distract themselves, by playing games on their phones and listening to music. Many suggested that sensory kits would be helpful, with items like earplugs, stress balls, fidget toys, play dough, and weighted blankets.

Almost all of the participants reported being left alone for long periods of time, which led to a “build-up” (P7) of anxiety. Several noted that they felt this lack of supervision was “strange” given the nature of their presentations, with one participant recalling,

*“[The staff member] closed the door and they were just like, ‘Stay here’… I thought for sure after doing something like that, I’d at least have someone there or someone talking to me or someone just to make sure that I’m okay before leaving the room, so I don’t do anything else.”*

(P3)

Indeed, one participant recalled attempting to engage in further self-harm when unsupervised. Overall, participants felt that they did not benefit from seeking help from the ED, that “not enough happened” (P7), and they “should have just stayed at home” (P10). One participant also reported being told by staff that “we can’t really do much” (P4), which deterred future help-seeking, and left participants with a sense of hopelessness. For some participants, the ED did not fulfil its expected therapeutic function, but instead was depicted as a holding place, “just somewhere for me to be” (P11), rather than a service that provides care. This led participants to feel unsafe:

*“It didn’t feel like a safe place for me, even though I knew I was at the hospital and there were doctors and everything there, it didn’t feel safe for me… physically yes, but emotionally or mentally, I didn’t feel safe at all. I felt really, really vulnerable.”*

(P3)

Several suggested that they had been discharged before they felt ready or safe to leave, which left them feeling “confused” (P8), with one stating, “I don’t know really why they let me [go] because I wasn’t okay.” (P7). This led to a sense of abandonment: “It was like, ‘You can go’, and that’s it.” (P6). Participants perceived there to be little thought about what would happen to them following discharge. Only four participants reported that their regular care providers had been provided with an update from the ED post-discharge. This was considered to be helpful:

*“They sent a discharge summary to my doctor, psychologist, and psychiatrist, and I always had that information in my wallet. That was good in the sense that I knew that they had the summary, and I didn’t have to worry about taking it to them.”*

(P5)

However, in most cases the young people had to be the one to inform their regular provider of their hospital presentation. All participants endorsed a preference for this communication to occur between services. Some reported that they had received follow-up phone calls from ED staff in the days following their presentation, but most did not. One participant recalled disappointment where staff had stated that they would follow-up but failed to do so. All participants indicated that a follow-up call would have been appreciated and may have encouraged future help-seeking.

Several directly attributed subsequent self-harm to how their ED experiences made them feel. One participant shared that their experience in the ED led them to promise themselves that “next time, I’m not even going to have a chance” (P9) of surviving their suicide attempt. Others reported that they concluded from their experience that they would need to engage in more serious/dangerous self-harm in future, to convey the severity of their distress. Adding further complexity, some participants discussed how their initial experiences deterred future help-seeking unless their self-harm was life-threatening. As a result, participants recognized that, as their subsequent presentations to the ED tended to be more dangerous, these presentations also tended to result in increased workload for staff. Thus, participants felt that “a vicious cycle” (P2) was formed, which was considered avoidable if adequate and appropriate care was provided in the first instance:

*“I wasn’t really left with many options, so I went home. Then a week later, I had a suicide attempt and was admitted to a psychiatric unit, which I feel like could have been prevented if I was not dismissed in the first place and was maybe given some options, as well, to help and not kind of left on my own to figure it out.”*

(P2)

#### 3.2.3. Staff Were Perceived to Be Disinterested, Dismissive, and Lacking in Knowledge

Participants perceived a lack of care, warmth, or empathy from most ED staff, describing them as “cold” (P8) and showing “no emotion at all” (P3). Most participants could not recall staff introducing themselves. This led to feelings of discomfort, with several participants acknowledging that they lied or withheld information for this reason:

*“I said, ‘This was just a one-off case where everything just got too much.’ But I knew inside that I had been dealing with this for a long time, but I didn’t really want to talk to her about it. She didn’t come across as a person that I would want to talk to in any situation.”*

(P3)

In contrast, when staff did show care and concern, through “even something as simple as tone” (P5) or “a kind face” (P11), participants felt more at ease and optimistic: “If I feel like I’m being talked to as a human, then I’m like, alright, I actually believe that things will be okay.” (P1). Some participants expressed a preference for staff to engage in humor to “make it a bit light-hearted” (P10), although another felt that this was inappropriate given the circumstances: “She was smiling, she was just like, ‘Oh, you tried committing suicide?’ and it was like, why are you smiling?” (P4).

Generally, staff were described as rushing through their assessments in a “robotic” (P8) way, which resulted in participants feeling overwhelmed by being “suddenly just thrown all these questions about what’s happened” (P7), particularly given their vulnerable mental state. Participants reported that there appeared to be little communication between staff, and noted the “tiring” (P7) and “overwhelming” (P13) process of being asked the same questions and “continuously saying the same thing over and over again” (P4). This was particularly difficult because participants’ stories were often “hard to talk about” (P5), which they felt staff did not grasp. Participants reported feeling like “you’re just like a number to them… [staff] want to be in and out” (P13). This deterred future help-seeking:

*“I’d come in for attempted suicide, but [staff acted like] it wasn’t that big of a deal… that was an insanely big thing in my life and for them it was just sort of, ‘Oh yeah, another person, another day’. That’s what’s going to happen again if I ever go in for attempted suicide, that’s how it’s going to be. The doctors and nurses there are going to act like they don’t care.”*

(P3)

Participants reported that staff implied their presentation was burdensome—that their presence was negatively impacting upon staff capacity to care for other, purportedly more serious cases:

*“She was making me feel as if I was wasting her time and that other people who had actually been injured and really needed their help were not getting what they needed because of me, and it was my fault.”*

(P8)

Indeed, a perception of ‘wastefulness’ was commonly expressed, with participants stating they felt like they were “wasting taxpayers’ money” (P9) and “wasting a bed” (P11), which induced guilt. Two participants also recalled how guilt was explicitly elicited by staff, specifically with regard to the impact of participants’ presentations on their parents—for example: “They were saying, ‘Do you feel bad that your Mum’s going through this?’ I said, ‘Yeah, I do. I don’t want to see her like this’.” (P12). In addition to the passive disinterest displayed by some staff, actively dismissive, invalidating and minimizing responses were also reported. This included staff commenting that participants’ presentations were “not that bad” (P10) or that their wounds were “just superficial” (P5). Participants felt like the legitimacy of their presentations was being questioned:

*“A nurse came in and I always remember this—she came in and she shook me, and she was like, ‘You can get up, we all know that what you took doesn’t make you tired.’ Then she walked out. I felt so attacked.”*

(P3)

Staff were also reported to ignore or disregard participants’ concerns, even when these were communicated explicitly, leading participants to feel unheard:

*“I told them, ‘I feel like if you send me home, I’m going to do something again.’ They told me straight up, ‘I don’t think you will.’… I felt like no-one was really hearing me, hearing what I really needed.”*

(P1)

Indeed, participants felt that presenting for help was seen as inherently indicative of lower risk by staff. Participants perceived that their distress did not “meet a threshold of what [staff] require” (P6); in falling short of this threshold, participants “couldn’t even be sick properly” (P2). Participants were also left frustrated by a feeling that staff were underestimating their distress as a result of them successfully regulating their behavior:

*“They’ve said, ‘Well you’re not screaming and throwing tantrums… so you can’t be that unwell’… if you were trying to be decent and not have to need restraints or any medication, it was that you’re not sick enough to be there. I feel like if you are presenting to ED for self-harm or any mental health thing in general, it is a crisis. But their idea of crisis seems to be that you have to be refusing help, not wanting it.”*

(P2)

Some expressed a belief that staff had received minimal or no training on self-harm, and that they were “really uneducated on adolescent mental health in general.” (P2). Staff were reported as seeming as though they “didn’t know what they were doing or talking about” (P8), and as a result “they don’t really do much with you” (P9). This was expanded upon by another participant, who felt that staff did not recognize that self-harm itself could be a young person’s way of indicating underlying distress:

*“That’s why people do these things [engage in self-harm]. They don’t know how to seek and ask for the appropriate help. And in a way these emergency departments, they have the opportunity to really ask them what they want and need.”*

(P9)

A number of participants believed that their age contributed to the minimization of their presentations, reporting that staff told them it was “just a phase” (P12), “just me being a bit dramatic” (P3), or that “heaps of people your age do this, it’s normal, you’ll get over it when you’re older” (P2). These responses felt “patronizing” (P2), which was further aggravated by the perception that staff took their presentations “a little bit more seriously” (P9) when they were accompanied by their parents. Some reported that their family members’ presence “had an effect on how nice [the staff] were.” (P8). Additionally, some participants felt that having relatives attend was useful, as they were better able to advocate for and communicate on the young person’s behalf, especially when “I wasn’t in a place to communicate well.” (P5). However, others reported that the involvement of their parents or relatives was distressing, and detailed instances of staff failing to respect their wishes on family involvement, particularly if they were still a minor:

*“When my parents did come, I asked that they weren’t let into the room straight away, just give me some time before they came in. But they said that they couldn’t do that… they let them in straight away which was really, really overwhelming for me… They said because I’m under 18 [and] my parents want to see me, they have to let them in.”*

(P3)

The impact of staff actions (and/or inaction) was magnified by participants’ already-negative mindset when they initially presented, as emphasized by one participant:

*“You’re distressed about whatever has made you distressed in the first place, plus the experience that you’ve had. Nothing should be adding to you feeling bad. If anything, it should be taking away.”*

(P2)

Participants recognized the context of a busy workplace and high caseload for staff, but expressed a desire that staff recognized the emotional context for attendees—that they “would realize that these people are coming from a vulnerable state and it is your job to provide them the space and the opportunity to get what they need from the emergency department.” (P9). When staff engaged in active listening, took their time, and endorsed that presenting was appropriate, participants felt reassured that their stories had been heard, and that their feelings “mattered” (P8):

*“A hospital environment is just naturally busy, staff have a million and one things to do, but with that worker it wasn’t obvious that they had to be in five places at once. It didn’t feel like that. So, that’s why it felt validating.”* (P6).

### 3.3. Safety, Feasibility, Acceptability

#### 3.3.1. Participants’ Levels of Pre- and Post-Participation Distress

The mean total score on the PHQ-4 was 5.85 (SD = 3.64), which indicated over the two weeks prior to participation in the interview, participants had been experiencing mild to moderate distress. A summary of participants’ responses to the questions assessing post-participation distress can be seen in Table 1. All participants stated that they felt participation was worthwhile; some participants expanded on this: “Feel like I’ve helped people and made something positive from a negative past experience”, “It will help other young people to feel more safe in that environment”, and “Research helps make improvements for the future”.

#### 3.3.2. Feedback on Methodology and Recommendations for Future Research on This Topic

Participants’ responses to questions seeking feedback on methods used to conduct research of this kind with young people are presented in Table 2. Participants were also given the opportunity to share other suggestions; the only suggestion that was offered was that “instead of all the physical paperwork, have it on an iPad, so it’s less confronting”; the participant was referring here to the paper consent form and pre- and post-interview questionnaires.

## 4. Discussion

The current study addressed a critical gap in the literature by exploring the experiences of young people who had presented to ED seeking care for self-harm. Participants recalled tangible details of their time in the ED, such as specific interactions with staff, and also described the emotional experience of presenting. They provided key insights into aspects of their treatment that they found both helpful and unhelpful, which may help shape future service provision.

### 4.1. Key Findings

Participants’ accounts of their experiences were overwhelmingly negative. This included sensory discomfort with aspects of the physical environment of the ED, aligning with previous Australian research [43]. Participants suggested the provision of sensory kits while waiting, to assist with distress regulation and distraction from rumination. Similar resources have previously been found effective in mental health inpatient care [44,45,46], and improvement of sensory experiences in the design and configuration of ED spaces have resulted in positive mental health outcomes [47,48]. All participants recounted waiting for extremely long periods of time, corroborating the findings of two reports commissioned by the Australasian College of Emergency Medicine [49,50]. Participants indicated that a perceived lack of privacy in ED waiting rooms affected the level and nature of the information that they provided at triage, which in turn may have resulted in an underestimation of their risk. They suggested being given a choice to provide triage-relevant information in a written format instead of verbally, potentially via a tablet device. Electronic tools have been implemented in other settings where young people receive mental health care, and have found to improve person-centeredness and increase disclosure and efficiency [51,52] and could readily be trialed in the ED.

Participants reported that most of their interactions with staff were perfunctory, characterized by dismissiveness and a lack of compassion. Indeed, participants’ accounts suggested that staff responses were underpinned by significant stigma about self-harm. Although staff themselves were not interviewed in the current study, previous studies with ED staff have consistently reported the presence of stigmatizing beliefs regarding self-harm [16,53,54,55]. Indeed, while this was the first study that directly asked young people about their experiences in the ED, the findings reveal that their experiences were not fundamentally different to those of adults [16]. However, in the current study, some participants perceived additional stigma associated with their age, such that their youth led staff to consider their self-harm less seriously. Participants reported being told “it’s just a phase”, echoing the misconception that people simply ‘grow out of’ self-harm, previously reported to be held by healthcare staff [56]. Researchers have raised concerns that this belief suggests that engagement in self-harm may be a normative stage of development [57,58,59,60,61,62]. Although, in many cases self-harm may remit [63], it is associated with a broad range of adverse long-term outcomes including future self-harm, suicide, poor vocational outcomes, and substance misuse [1]. Participants’ accounts of “patronizing” responses from staff, such as being seen as “a bit dramatic”, suggest little recognition of the psychological and social problems faced by young people. ED staff may benefit from training on working with young people, as has been recommended for other clinicians such as general practitioners [64,65]. The provision of developmentally-sensitive services must also address the innate challenge of appropriately involving parents and other guardians in young people’s care [66]; as the findings of the current study indicate, parental accompaniment in the ED may be positively experienced by some young people and negatively by others.

The data highlighted a series of inherent contradictions; for example, participants perceived that staff believed help-seeking itself was indicative of lower risk. As a result, participants communicated a belief that more serious/dangerous self-harm was required in order to receive adequate attention from staff. This refutes the view, reported in the literature, that negative treatment (including stitching wounds without the use of anesthetic) would serve as ‘punishment’ or ‘a lesson’, and thus deter future self-harm [67,68,69,70]. Instead, participants overtly attributed later, more dangerous self-harm to the emotional impact of their negative encounters in the ED. Moreover, they indicated these negative encounters in the ED deterred future help-seeking. This occurred through several mechanisms: participants felt they were a burden on the system, that staff did not care, and that staff could not do anything to help. It is likely that help-negation may have compounded these factors [71]. For these reasons, they reported only seeking help for subsequent episodes of self-harm when it was absolutely necessary. Given that those who seek help represent the minority of young people who self-harm [11], this avoidance of help-seeking is extremely concerning. It is critical that the healthcare system capitalizes on ED presentations as an indication of a young person’s desire for help, and therefore an opportunity for intervention and prevention of potential more lethal injuries.

### 4.2. Opportunities for Service Improvement

This study highlights several areas that require considerable improvement in order to provide appropriate and adequate care for young people seeking help from EDs for self-harm.

#### 4.2.1. Staff Training

The findings reveal the pressing need for staff education on self-harm and working with young people. Optimistically, systematic reviews demonstrate that receipt of training is associated with more positive attitudes among healthcare staff towards patients at risk for suicide [72,73]. Staff have previously identified a need for psychoeducation on self-harm and the assessment of risk and protective factors [54,74]. Training should emphasize the importance of person-centered care, including demonstrating warmth, empathy, active listening, and timely communication of information [75,76,77,78,79]. Importantly, participants in the current study stressed that they did not expect to be prioritized or to receive special provisions, but merely sought to receive the same treatment as others.

#### 4.2.2. Aftercare

The period after discharge from the ED is a high-risk time for young people who have presented with self-harm, with high likelihood of re-representation to ED [80,81]. Participants in the current study reported feelings of being abandoned at discharge, with no consideration of their ‘next steps’ for longer-term support, and few receiving any kind of follow-up. Discharge procedures have been raised as an issue in previous studies with adults who sought care for self-harm from EDs [16]. Participants advocated for the provision of a treatment summary at discharge, to facilitate communication with their regular care providers. It is critical that aftercare is integrated into standard crisis and acute care provision, including referral and follow-up [82,83]. Assertive outreach programs, such as the Hospital Outreach Post-suicidal Engagement (HOPE) initiative in Victoria [84], are currently being piloted and show promise, but youth-specific versions that can be delivered at scale and rigorously evaluated are urgently required.

#### 4.2.3. Service Reform

Most significantly, this study substantiates the need for mental health service reform. Participants in the current study were recruited through headspace, and were thus receiving care. Furthermore, the study did not assess whether their initial presentation to ED represented their first interaction with mental health services. However, service gaps in the broader mental health system are well-established [85,86], and as a consequence, EDs serve as a source of care for those who require immediate mental health support, particularly after hours [50]. Public health messaging emphasizes contacting emergency services and presenting to ED when experiencing suicidal crisis, yet EDs have not been designed or resourced to meet these needs. This has led to calls for alternative models of care [87,88], which have received investment and are being trialed in Australia and elsewhere in the world [89]. However, the evidence base for appropriate and effective alternatives is currently limited. Further evaluation is required, as are efforts to ensure that the introduction of these alternatives does not further fragment an already-fragmented system.

Some participants’ episodes of self-harm involved suicidal intent, while others did not. EDs need to be able to respond compassionately and effectively to self-harm, regardless of intent, remaining cognizant that self-harm is one of the most consistent known risk factors for suicide [90]. Furthermore, it is critical that services recognize that, although young people seeking care for self-harm may present with physical injuries, these are driven by underlying psychological distress. It is therefore essential that the broader system of care meets young people’s mental health needs upstream, at an earlier stage in their trajectory before crisis occurs. Within Australia, the State of Victoria is currently engaged in transformational service reform through the Royal Commission into Victoria’s mental health system [88], which provides a unique opportunity to learn from young people’s concerns, and implement meaningful change.

### 4.3. Acceptability, Feasibility and Appropriateness of Conducting Research on This Topic with Young People

This study provides valuable information for researchers regarding the safety of asking young people about their experiences of care for self-harm. Participants’ distress before and after participating in the interview was measured, and the results indicate that participation in the study did not induce distress. This may have been achieved by the conduct of the study, which was guided by practices drawn from literature on trauma-informed clinical care, and sensitive inquiry in qualitative research [29,91,92]—see Supplementary File S6 for more details. Future research with similar participants may similarly benefit from implementation of these practices. In particular, the approach to data collection aimed to avoid the repetition of behaviors that participants in previous studies reported as distressing when exhibited by ED staff, such as inattentiveness, impatience, dismissiveness, and vagueness in communication [16,22]. In contrast, we aimed to demonstrate empathy and respect through providing clear explanations, reflecting and re-stating participants’ responses to check the accuracy of our understanding, and allowing participants’ time to gather their thoughts. Additionally, throughout the interview, the researchers sought to validate participants’ responses.

### 4.4. Strengths and Limitations

The main strength of this study was its foregrounding of young people’s voices, especially those who are often excluded from research based on perceived risk [93,94]. Although the importance of patient and public involvement is increasingly recognized, few studies in suicide prevention feature active partnerships with young consumers [95].

Limitations of the study primarily relate to the sample. Participants in this study were recruited through headspace centers, and thus were currently engaged with ongoing support. While this was an important component of the study’s safety protocol, it means that participants may not be representative of all young people who seek care for self-harm from EDs, in particular those who are discharged without further follow-up. Participants with multiple experiences in the ED were invited to focus on one experience that they remembered particularly well; it is possible that participants may have chosen an experience that was notably negative [96,97]. Additionally, young people who wished to report negative feedback may have been more motivated to participate in the study, together resulting in the potential overrepresentation of negative accounts. Most of the participants were female; although young females are more likely to engage in self-harm than young males [98], the findings of the current study may not be reflective of the experiences of young males or other genders. None of the participants identified as Aboriginal or Torres Strait Islander. As suicide rates among Aboriginal and Torres Strait Islander young people are considerably higher than their non-indigenous counterparts [99,100], it is critical that their experiences are captured in future research.

## 5. Conclusions

This study addresses a critical gap in the literature by exploring young people’s experiences of seeking care for self-harm from EDs. While careful to acknowledge the systemic issues that impact EDs, such as lack of time, space, and other resources, participants were often left frustrated and disappointed as services failed to meet their needs, or indeed, in some cases, actively increased their distress and risk. This represents a critical missed opportunity to provide care to vulnerable young people at a time of heightened risk. Whilst many of the limitations associated with care received in the ED underline the need for widespread service reform, many simply reflect the need for a compassionate and person-centered response, which is neither costly nor time-consuming.

The study also demonstrates that it is both safe and acceptable to conduct research of this nature; indeed, we would argue that it is unacceptable not to. Young people with lived experience of suicide and self-harm are routinely excluded from research, yet they have extremely valuable insights that have the potential to contribute to both future research, and to service reform.

## Figures and Tables

**Table 1:** Summary of participants’ responses to questions assessing post-participation distress.

Item	% of Sample
	‘Mostly’ or ‘strongly’ agree
I’m glad I participated in this study	84.6
This study was interesting	76.9
I would be willing to participate in similar studies in the future	76.9
	‘Mostly’ or ‘strongly’ disagree
Participating in this study was distressing	76.9
Participating in this study upset me	76.9
Participating in this study brought up unpleasant thoughts and feelings	84.6
	The event described would be worse than this study
Forgetting Mothers’ Day	61.5
Losing $20	76.9
Spilling coffee on a new shirt	84.6
Being alone at a party	100
Having blood drawn	61.5

**Table 2:** Summary of participants’ responses to questions seeking feedback on methodology.

Item	% of Sample
**Optimal time to contact a young person to speak about their experience following presentation to ED**	
One week post-presentation	38.5
One month post-presentation	28.5
Longer than a month	15.4
Other: “When they feel ready”	7.7
**Best way to contact a young person to take part in this type of research**	
By text message	53.8
By phone call to their mobile phone	38.5
By email	7.7
**Best way to ask a young person about their experiences in ED**	
Face-to-face interview	61.5
Survey	15.4
Other: “Option of survey or interview”	15.4
Other: “Interview with someone at headspace they trust or let them write out the answers”	7.7

## Data Availability

The data presented in this study are available on request from the corresponding author. The data are not publicly available due to ethical restrictions.

## Supplementary Materials

The following are available online at https://www.mdpi.com/1660-4601/18/6/2892/s1, S1: COREQ checklist, S2: Participant Information and Consent Form, S3: Demographics questionnaire, S4: Interview schedule, S5: Feedback on methodology questionnaire, S6: Safety practices.
