# Supplementary material for: A Qualitative Account of Young People’s Experiences Seeking Care from Emergency Departments for Self-Harm
Source: Int J Environ Res Public Health. 2021 Mar 12;18(6):2892. doi: 10.3390/ijerph18062892 (PMC8000083; doi:10.3390/ijerph18062892)
Supplement: Supplementary file 1 [file ijerph-18-02892-s001.zip › ijerph-1110374-supplementary/Supplementary Files/S2 Participant Information and Consent Form.docx]

PARTICIPANT INFORMATION and consent form

**The University of Melbourne/Orygen, the National Centre of Excellence in Youth Mental Health**

Participant Information and Consent Form for Young People

Version: 2

Dated: February 2019
Site: Orygen / headspace

**Full Project Title:** Consumers’ experiences seeking help from an emergency department for self-harm: An initial pilot study

**Principal Researcher:** Sadhbh Byrne

**Associate Researcher(s):** Dr Jo Robinson, Dr Simon Rice, Dr Sarah Bendall, Michelle Lamblin, Nina Stefanac, India Bellairs-Walsh, Dr Sophie Adams, Dayna Minovski, Alex Pleban, Meghan O’Keefe, Brianna McGregor, Emily Boubis

This Participant Information and Consent Form is 9 pages long. Please make sure you have all the pages.

1. Your Consent

You are invited to take part in this research project.

This Participant Information sheet contains detailed information about this research project. Its purpose is to explain to you and your parent/guardian as openly and clearly as possible all the procedures involved in this project before you decide whether or not to take part in it.

Please read this information carefully. Feel free to ask questions about any information in the document. You may also wish to discuss the project with a relative or friend or your local health worker. Feel free to do this.

Once you understand what the project is about, and if you agree to take part in it, you and your parent/guardian (if under 18) will be asked to sign the Consent Form. By signing the Consent Form, you indicate that you understand the information and that you give your consent to participate in the research project.

You will be given a copy of the Participant Information and Consent Form to keep as a record.

2. Purpose and Background

Background:

A review was recently published which examined all the evidence currently available on self-harm presentations to emergency departments (EDs). This review found that there is a substantial gap in our knowledge of self-harm presentations to EDs, specifically the perspectives and experiences of young consumers. This means that young people’s voices are missing from our current understanding of optimal treatment of self-harm in the ED.

In order to address this gap, we are conducting this pilot study to establish a protocol for larger-scale study which will examine young consumers’ experiences of seeking care for self-harm for EDs.

Purpose:

You are invited to take part in this project because you:

- are currently engaged in treatment at headspace Glenroy, headspace Werribee, headspace Craigieburn, or headspace Sunshine;

*and*

- you have previous experience presenting to an ED with any self-inflicted physical injury, with or without suicidal intent.

We would like to hear about your experiences, and any suggestions you have about how care received from EDs can be improved. Your feedback will help guide the development of a protocol for a future larger-scale research study.

3. Procedures

If you agree to take part, we will ask you to complete a Wellness Plan in advance. This plan has been developed through consultations with young people, with the aim of helping to ensure that we can best support you throughout your participation in this project. The plan includes an emergency contact, topics you might find distressing, signs of distress that the researcher can look out for, and things that help you when you feel distressed (for example, listening to music).

We will then arrange a time for you to meet a member of our research team at a headspace centre for your participation session.

During the participation session, the researcher will firstly ask you to complete a short pre-interview questionnaire about how you have been feeling over the previous two weeks. This is called the Participant Pre-Interview Questionnaire.

You will then be asked to participate in a one-on-one interview, which will be conducted by a member of the research team, and audio-recorded. You will be asked questions such as “Starting at the point of your arrival, could you describe what happened during your time in the emergency department? For example, who you spoke to, or what they asked?”

Upon completion of the interview, the researcher will ask you to complete a short feedback form about the study.

You will then be asked to complete a short questionnaire about how taking part in the interview made you feel. This is called the Participant Post-Interview Questionnaire.

Lastly, you will complete a mood-elevating activity. Where possible, we will use some of the things you mentioned in your Wellness Plan – for example, we might listen to some music. Alternatively, we might do a short mindfulness meditation session, or watch a relaxing nature video. Other people who have taken part in research on sensitive topics like self-harm have found completing a mood-elevating activity helpful. However, you do not have to take part in this activity – you can tell the researcher if you would rather not complete this part.

It is anticipated that the entire process will last approximately 1 hour and 15 minutes, of which 45 minutes will be allocated to the interview.

4. Possible Benefits

A key advantage of taking part in this research will be your ability to provide valuable feedback, that will inform the development of a protocol for a future larger-scale study examining consumers’ experiences seeking care for self-harm in EDs. However, we cannot guarantee any benefits from taking part. What we do hope is that the information we collect will allow us to suggest ways in which the care young people receive from EDs could be enhanced, which in turn is expected to improve the experiences of young people in the future.

5. Possible Risks

There are no physical risks associated with being in this study.

However, there may be unforeseen or unknown risks, or you may become distressed as a result of talking about self-harm.

If you feel upset while participating in the interview, please let the researcher present know. If necessary, the research team can arrange additional support for you. This may include contacting your nominated support person listed in your Wellness Plan, and/or arranging additional support for you from your doctor or headspace clinician.

You can change your mind and decide you do not wish to take part in the study at any time during the participation session with the researcher. This will **not** affect in any way your involvement with services.

You can also ask for your project records to be destroyed or erased after the participation session. If you do this, we will securely destroy or erase your information.

There is one situation where we will not be able to destroy or erase some of your information. On page 4 of this form, we explain that your answers on the Participant Pre-Interview Questionnaire and Participant Post-Interview Questionnaire will be posted to the Open Science Framework public repository. We will not be able to remove your answers from the Open Science Framework public repository after the answers have been posted, because it will not be possible to identify which answers are yours.

6. Alternatives to Participation

You do not have to participate, and it is important for you to know that if you decide not to participate this will **not** affect in any way your involvement with services.

If you decide to take part, you can later change your mind and withdraw from the project at any time during the participation session.

7. Privacy, Confidentiality and Disclosure of Information

Any identifying information we collect about you will remain confidential. It will only be disclosed with your permission, or as required by law and for safety concerns. If this is the case and the research team are concerned about you, then your parent, guardian, or nominated support person, and your headspace clinician, will be contacted. If this does occur, you will be consulted.

We will securely store your information, including audio recordings of interviews, in password-protected files on password-protected computers. Any information collected in hard-copy (i.e., on paper) will be stored in a locked filing cabinet at Orygen. We will securely store these files for an indefinite period of time.

| **We will post the answers (e.g. which rating you selected from 1 to 5) you provide on the Participant Pre-Interview Questionnaire and Participant Post-Interview Questionnaire to the Open Science Framework public repository.** The reason we will do this is so that these answers can be compared to the answers given by other people who take part in similar research studies. By posting the answers to this repository, the data will be declared as public domain.  **None of the information collected on these two questionnaires will personally identify you. None of the other information you provide will be posted to this depository.**  **We will not be able to remove your answers from the Open Science Framework public repository after the answers have been posted, because it will not be possible to identify which answers are yours.** |
| --- |

We plan to present the findings from this project in a report, as well as written articles in scientific journals or presentations at scientific conferences. There will be no way of identifying any individual in the project in these reports, articles, or presentations.

*How you can access the information:*

The only information we will have about you is what you tell us. Should you want to see this information, you can ask a member of the research team and it will be made available to you.

8. New Information Arising During the Project

During the research project, new information about the risks and benefits of the project may become known to the researchers. If this occurs, you will be told about this new information. This new information may mean that you can no longer participate in this research. If this occurs, the person(s) supervising the research will stop your participation. In all cases, you will be offered all available care to suit your needs and medical condition (if relevant).

9. Results of Project

Results from the study will be available in a final study report, and also via the journals in which they are published, copies of which will be made available to you on request.

If you would like to be provided with some feedback on how your responses informed the development of the protocol for the larger-scale study, there is a space on the attached consent form where you can write your email address. We will use this email address solely for the purpose of contacting you with the feedback once this pilot study has been completed.

If you have any questions at any time during the study, please feel free to contact the principal researcher on the phone numbers or e-mail below. You can ask the researcher carrying out the interview any questions which you may have. If you would like more information about the study, or if you have some concerns about it, either now, or in the future, do not hesitate to contact one of the researchers involved.

10. Further Information or Any Problems

If you require further information or if you have any problems concerning this project, you can contact the principal researcher or any of the research team. The researcher responsible for this project is **Sadhbh Byrne – mobile: 0428 815 170 (available to receive calls Monday to Friday, 09:00 – 17:00), email:** [**sadhbh.byrne@orygen.org.au**](mailto:sadhbh.byrne@orygen.org.au)**.**

If you are in distress and need urgent support, you can contact **Orygen Youth Health triage** at **1800 888 320**.

11. Other Issues

If you have any complaints about any aspect of the project, the way it is being conducted or any questions about your rights as a research participant, then you may contact

Position: Executive Officer, Human Research Ethics, The University of Melbourne

Telephone: (03) 8344 2073

You will need to tell the officer the name of the researcher given in section 10 above.

12. Participation is Voluntary

Participation in any research project is voluntary. If you do not wish to take part, you are not obliged to. If you decide to take part and later change your mind, you are free to withdraw from the project at any stage during your participation session. If you choose to not contribute all or part of the information requested, this will have no effect on your treatment at headspace or your relationship with Orygen or headspace.

Before you make your decision, a member of the research team will be available to answer any questions you have about the research project. You can ask for any information you want. You may also wish to discuss this project with a friend, family member or other person. If so, you should feel free to do this. Sign the Consent Form only after you and your parent/guardian have had a chance to ask your questions and have received satisfactory answers.

If you decide to withdraw from this project, please notify a member of the research team before you withdraw. This notice will allow that person or the research supervisor to inform you if there are any health risks or special requirements linked to withdrawing.

13. Ethical Guidelines

This project will be carried out according to the *National Statement on Ethical Conduct in Research Involving Humans* (June 2007), produced by the National Health and Medical Research Council of Australia. This statement has been developed to protect the interests of people who agree to participate in human research studies.

The ethical aspects of this research project have been approved by the University of Melbourne Human Research Ethics Committee. The project is being funded by Future Global Generations and the William Buckland Foundation.

CONSENT FORM
(Attach to Participant Information)

The University of Melbourne/Orygen

Consent Form Version: 2

Dated: February 2019
Site: Orygen/headspace

**Full Project Title:** Consumers’ experiences seeking help from an emergency department for self-harm: An initial pilot study

**Principal Researcher:** Sadhbh Byrne

**Associate Researcher(s):** Dr Jo Robinson, Dr Simon Rice, Dr Sarah Bendall, Michelle Lamblin, Nina Stefanac, India Bellairs-Walsh, Dr Sophie Adams, Dayna Minovski, Alex Pleban, Meghan O’Keefe, Brianna McGregor, Emily Boubis

I have read and I understand the Participant Information version 1, dated February 2019.

I freely agree to participate in this project according to the conditions in the Participant Information statement.

I understand that my involvement in the project is for research purposes, and is voluntary. I understand that I am free to withdraw at any time, and free to withdraw any unprocessed identifiable data previously supplied.

I understand that any identifying information collected about me will remain confidential and will only be disclosed with my permission, or as required by law and for safety concerns.

I will be given a copy of the Participant Information and Consent Form to keep.

Participant’s Name (printed) ………………………………………….

Signature Date

If you would like to be emailed the findings from this project, explaining how your responses helped to inform the development of the protocol for the larger-scale study, please supply your email address below.

Email: ……………………………………………………

Researcher’s Name (printed) ……………………………………………………

Signature Date

*Note:* All parties signing the Consent Form must date their own signature.

THIRD PARTY CONSENT FORM

*(To be used by parents/guardians of minor children – under 18.)*

(Attach to Participant Information)

The University of Melbourne/Orygen

Third Party Consent Form
Consent Form Version: 2

Dated: February 2019
Site: Orygen/headspace

**Full Project Title:** Consumers’ experiences seeking help from an emergency department for self-harm: An initial pilot study

**Principal Researcher:** Sadhbh Byrne

**Associate Researcher(s):** Dr Jo Robinson, Dr Simon Rice, Dr Sarah Bendall, Michelle Lamblin, Nina Stefanac, India Bellairs-Walsh, Dr Sophie Adams, Dayna Minovski, Alex Pleban, Meghan O’Keefe, Brianna McGregor, Emily Boubis

I have read and I understand the Participant Information version 2, dated February 2019.

I give my permission for _____________________ to participate in this project according to the conditions in the Participant Information.

I understand that this project is for research purposes, is voluntary and that the participant is free to withdraw at any time, and free to withdraw any unprocessed identifiable data previously supplied.

The researcher has agreed that the participant’s identity and personal details will remain confidential and will only be disclosed with their permission, or as required by law and for safety concerns.

I will be given a copy of Participant Information and Consent Form to keep.

Participant’s Name (printed) ……………………………………………………

Name of Person giving Consent (printed) ……………………………………………………

Relationship to Participant: ………………………………………………………

Signature Date

*Note:* All parties signing the Consent Form must date their own signature.

REVOCATION OF CONSENT FORM

*(To be used for participants who wish to withdraw from the project.)*

(Attach to Participant Information)

The University of Melbourne/Orygen

Revocation of Consent Form

**Full Project Title:** Consumers’ experiences seeking help from an emergency department for self-harm: An initial pilot study

**Principal Researcher:** Sadhbh Byrne

**Associate Researcher(s):** Dr Jo Robinson, Dr Simon Rice, Dr Sarah Bendall, Michelle Lamblin, Nina Stefanac, India Bellairs-Walsh, Dr Sophie Adams, Dayna Minovski, Alex Pleban, Meghan O’Keefe, Brianna McGregor, Emily Boubis

I hereby wish to WITHDRAW my consent to participate in the research proposal described above and understand that such withdrawal WILL NOT jeopardise any treatment or my relationship with ORYGEN.

Participant’s Name (printed) …………………………………………………….

Signature Date
