# Supplementary material for: A Qualitative Account of Young People’s Experiences Seeking Care from Emergency Departments for Self-Harm
Source: Int J Environ Res Public Health. 2021 Mar 12;18(6):2892. doi: 10.3390/ijerph18062892 (PMC8000083; doi:10.3390/ijerph18062892)
Supplement: Supplementary file 1 [file ijerph-18-02892-s001.zip › ijerph-1110374-supplementary/Supplementary Files/S3 Demographic Sheet.docx]

**
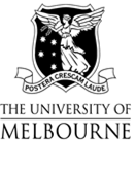
**

**Consumers’ experiences seeking help from an emergency department for self-harm:**

**An initial pilot study**

**Participant demographic sheet**

**Demographic Information**

(Please mark the corresponding box)

**1.** Age: ______ years

**2.** I identify as (Please tick all that apply):

🞏_1_ Male

🞏_2_ Female

🞏_3_ Intersex, trans, gender fluid, or gender diverse

🞏_4_ Prefer not to say

🞏_5_ Other (please specify: ___________________)

**3.** Were you born in Australia? (Please mark the corresponding box)

🞏_1_ Yes

🞏_2_ No (please specify where you were born: _____________________)

**4.** Do you identify as Aboriginal / Torres Strait Islander? (Please mark the corresponding box)

🞏_1_ Yes

🞏_2_ No

**5.** Is English the main language spoken in your home? (Please mark the corresponding box)

🞏_1_ Yes

🞏_2_ No

_____________________________________________________________________
