# Supplementary material for: A Qualitative Account of Young People’s Experiences Seeking Care from Emergency Departments for Self-Harm
Source: Int J Environ Res Public Health. 2021 Mar 12;18(6):2892. doi: 10.3390/ijerph18062892 (PMC8000083; doi:10.3390/ijerph18062892)
Supplement: Supplementary file 1 [file ijerph-18-02892-s001.zip › ijerph-1110374-supplementary/Supplementary Files/S4 Interview Schedule.docx]

**Consumers’ experiences seeking help from an emergency department for self-harm:**

**An initial pilot study**

**
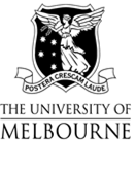
**

**Principal researcher:** Sadhbh Byrne

**Ethics ID number:** 1852466.1

**Date:** 03/10/2018

**Version number:** 1

**Interview Schedule**

| **Questions** | **Additional Prompts** |
| --- | --- |
| *If participant was recruited through Stream Two:*  Could you tell me when (approximately) you presented to an emergency department seeking care for self-harm? |  |
| Starting at the point of your arrival, could you describe what happened during your time in the emergency department? | For example, who you spoke to, what they said or asked?  What was your experience in the waiting room?  Were you brought to another room? What was that room like? |
| Were there any positive aspects to this experience? | If so, could you please describe them? |
| Were there any negative aspects to this experience? | Was there anything about your experience you would like to have been different?  If so, could you please describe them? |
