# Supplementary material for: A Qualitative Account of Young People’s Experiences Seeking Care from Emergency Departments for Self-Harm
Source: Int J Environ Res Public Health. 2021 Mar 12;18(6):2892. doi: 10.3390/ijerph18062892 (PMC8000083; doi:10.3390/ijerph18062892)
Supplement: Supplementary file 1 [file ijerph-18-02892-s001.zip › ijerph-1110374-supplementary/Supplementary Files/S5 Participant Study Feedback Form.docx]

**
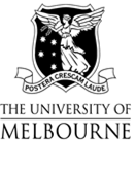
**

**Consumers’ experiences seeking help from an emergency department for self-harm:**

**An initial pilot study**

**Participant feedback questionnaire**

**Please place an X in the box that corresponds with your answer. You may also use the space provided to explain your answer further, if you would like.**

**1.** Do you feel that your participation in this research study was worthwhile?

🞏_1_ Yes

🞏_2_ No

If you would like to explain your answer further, please do so here: __________________________________________________________________________________________________________________________________________

**2.** In your opinion, when would be the best time to contact someone about participating in a research study like this?

🞏_1_ The day after attending the emergency department

🞏_2_ A week after attending the emergency department

🞏_3_ A month after attending the emergency department

🞏_4_ After more than a month has passed since attending the emergency department

🞏_5_ Other – please explain:

______________________________________________________________________________________________________________________________

**3.** In your opinion, what would be the best way to contact a young person about participating in a research study like this?

🞏_1_ By text

🞏_2_ By e-mail

🞏_3_ By letter mailed to their home address

🞏_4_ By phone call to their mobile phone

🞏_5_ Other – please explain:

_____________________________________________________________________________________________________________________________________________________________________________________________

**4.** In your opinion, what would be the best way to ask young people about their experiences in the ED?

🞏_1_ One-on-one interview

🞏_2_ Survey

🞏_3_ Other – please explain:

__________________________________________________________________________________________________________________________________________________________________________________________

**5.** Do you have any other suggestions for how we could best carry out this research?

_______________________________________________________________________________________________________________________________________________________________________________________________________________________________________________________________________________________________________________________________________________________________________________________________________________________________________________________________________________________________________________________________________________________________________________________________________________________________________________________________________________________________________________________________________________________________________________________
