# Supplementary material for: A Qualitative Account of Young People’s Experiences Seeking Care from Emergency Departments for Self-Harm
Source: Int J Environ Res Public Health. 2021 Mar 12;18(6):2892. doi: 10.3390/ijerph18062892 (PMC8000083; doi:10.3390/ijerph18062892)
Supplement: Supplementary file 1 [file ijerph-18-02892-s001.zip › ijerph-1110374-supplementary/Supplementary Files/S6 Safety Considerations and Practices.docx]

A qualitative account of young people’s experiences seeking care from emergency departments for self-harm

**Supplementary File 6: Safety Considerations and Practices**

Each participant was asked to complete a Wellness Plan prior to their interview. This included personal and emergency contact details, topics the participant found particularly distressing, personal indicators of distress, and self-soothing strategies. These were reviewed by the researchers prior to each interview. We employed face-to-face interviews to facilitate researchers’ awareness to participants’ non-verbal cues of distress, and to ensure that the risk management protocol could be enacted in-person and in a timely manner if required. This protocol incorporated the information contained in participants’ Wellness Plans and, in the case of elevated risk, appropriate management by a clinician. As far as was possible, research assessments were scheduled immediately before the participant’s headspace appointment to ensure that they could check in with their treating clinician afterwards, and to enhance accessibility of participation. This also ensured that the participant was familiar with their surroundings. Prior to the commencement of the interview, time was allotted for rapport-building with the participant. Stress balls and fidget toys were provided for participants, as these have been reported to be useful by young people participating in other research from our team [1]. Throughout the interviews, both researchers employed active listening, monitored participant distress, and maintained rapport. Guidance for sensitive interviewing suggest that flexibility is key [2]. As such, while some broad questions had been prepared in the interview schedule, we endeavoured to enable the participants to determine the specific topics discussed.

The decision to have two researchers present in the interview was carefully considered, due to the inherent power imbalance between researcher and participant. In our experience, one-on-one interviews, especially with young people, can unintentionally amplify the perceived ‘authority’ of the researcher. This can be further exacerbated when the young person is seated directly across from the focused attention of the researcher. To address this and redistribute the perceived power, we arranged the three chairs in a circle, and actively pursued an informal tone throughout the interview, such that the three individuals present were each ‘participants’ in the conversation shared between them [3]. The presence of two researchers also served an additional goal of differentiating the interview from a therapeutic encounter, in which the young person would typically interact one-on-one with a clinician.

At the conclusion of the interview, the researchers asked each participant if they would like to complete a mood-elevating activity. This practice has been utilized in previous studies and found to be beneficial [4]. Activities included watching a relaxing nature video, completing a mindfulness meditation activity, and listening to the participant’s favorite song. Participants were provided with a debriefing sheet, which thanked them for their participation and provided contact details for online and telephone support services.

Throughout data collection, the two interviewers met regularly to debrief with a third member of the research team (S.R.), who provided additional supervision and debriefing opportunities as needed. This was to help mitigate any risk of mental health distress arising in the interviewers, and is recommended in suicide prevention research where mental well-being may be impacted from listening to sensitive and potentially distressing respondent stories [5].

**References**

1. Thorn, P., et al., *Developing a suicide prevention social media campaign with young people (The #Chatsafe project): co-design approach.* JMIR mental health, 2020. **7**(5): p. e17520.

2. Liamputtong, P., *Researching the Vulnerable: A Guide to Sensitive Research Methods*. 2007, London: SAGE Publications.

3. Karnieli-Miller, O., R. Strier, and L. Pessach, *Power relations in qualitative research.* Qualitative health research, 2009. **19**(2): p. 279-289.

4. Arbuthnott, A.E., S.P. Lewis, and H.N. Bailey, *Rumination and emotions in nonsuicidal self‐injury and eating disorder behaviors: A preliminary test of the emotional cascade model.* Journal of clinical psychology, 2015. **71**(1): p. 62-71.

5. Chen, J.I., G.L. Mastarone, and L.M. Denneson, *It's not easy–impacts of suicide prevention research on study staff*. 2019, Hogrefe Publishing.
